# Supplementary material for: Intra- and inter-individual variability in the underwater pull-out technique in 200 m breaststroke turns
Source: PLoS One. 2023 Mar 21;18(3):e0283234. doi: 10.1371/journal.pone.0283234 (PMC10030011; doi:10.1371/journal.pone.0283234)
Supplement: Attachment — Submitted filename: Responses to the reviewers_submit.docx [file pone.0283234.s002.docx]

Reviewer #1: GENERAL COMMENT
The present manuscript contains an interesting study about variations on the breaststroke pullout actions throughout a 200m breaststroke time trial. Authors make a thoughtful data analysis and present results in a proper document from a formal point of view. The main concern for the present reviewer is that results and discussion are difficult to follow due to terminology employed. Authors could make an effort to present coordination analysis in a more “intuitive” way. As a former swimming coach with experience in the analysis of breaststroke swimmers, the present manuscript is far from clearly extracting the main practical applications.
More specifically, in relation to time gaps during pull out actions, T1-T6 would be expected to be named in the chronological order they occur. Of course, there are different pull-out combinations between swimmers but literature has described the most common patters both in competition and experimental conditions. For example, T6 would usually occur the first time gap…why is then called T6? Considering all swimmers in the present sample performed dolphin kick before the arm pullout…why both T4 and T5 were included? For the simplicity sake,… could T5 be removed from analysis? (lines 277-283 are an example on how complicated this analysis gets)
A suggestion of time gaps in chronological order would be:
1. Dolphin kick to arm pullout
2. Dolphin kick to leg recovery
3. Arm pullout to arm recovery
4. Arm recovery to Leg recovery
5. Breaststroke kick to Arm stroke
In addition, it would be much more easy-to-understand if time gaps were named according to the key events they refer. For example, the “dolphin kick to arm pullout” or “DK-AP” gap instead of T1. Otherwise, it is difficult for readers to follow arguments in the manuscript even if they are used to the swimming terminology (because there are 6 time gaps, maybe with one or two would be easier).

Response: The authors appreciate the reviewer for spending time assessing the manuscript. When going through the responses, please note that the line information provided in this document all corresponds to the clean manuscript (not the track-changed version).

The authors agree that the terminologies made the text flow complex, therefore the terminologies have been updated, and a figure illustrating three clusters’ typical coordination patterns has been inserted. Regarding the terminologies, we followed the reviewer’s suggestions and named each time gap based on the events. At the same time, we kept the numerical names in square brackets too, as we felt it would perhaps make things clearer for readers who potentially want to compare the current manuscript with the authors’ past papers dealing with the same topic.

SPECIFIC COMMENTS
Line 57 is there any evidence on the role of inter-limb coordination in underwater swimming? Not sure the reference of Connaboy 2009 covers this type of coordination during UUS

Response: The coordination between the upper and lower body in UUS is essentially the wave characteristics (Phase angle and wave velocity), which is covered in the review by Connaboy et al. (2009). However, as the review paper by Connaboy focuses on a wide range of animals, using this reference to support the argument is probably a bit confusing – therefore, we replaced the reference with Elipot et al. (2010), which hopefully made the argument more straightforward. Furthermore, probably “Inter-limb coordination” might not be the most appropriate word to describe the wave characteristics in UUS. Therefore, the phrase was rephrased by “Inter-limb (or inter-segment) coordination” (L58-59) for clarity.

Line 171, despite previous studies of the same group provided a graphical illustration of pull out phases and time gaps, there is no graphical description in the present study. This undoubtedly would help readers to underwater what authors are measuring. At least, a clear reference to previous studies where a graphical description was included should be cited in the methods section.

Response: We agree that having a figure illustrating each time gap would be more helpful for readers. A figure similar to the previous studies has been included as Figure 1.

Line 197, it would be interesting to include the first breaststroke kick and subsequent arm pull on the analysis of the breaststroke underwater strategies.

Response: We agree that including more sequences, such as the first arm motion, would be interesting to investigate more about the transition phase. However, to include the first transition stroke in the analysis, it is essential to consider the coordination pattern during the surface swimming because without the surface swimming information, it would be difficult to conclude if the detected coordination patterns are specific to the turn phase or those are just how swimmers perform their surface swimming. Investigating the transition stroke together with the surface swimming technique would be a different research question from the aim of the present study. Due to those reasons, the authors would prefer to focus on the time gaps specific to the underwater segment. Despite the decision, the authors do agree that it is an important topic, and therefore it is highlighted as a future suggestion at the end of Discussion (L474-477).

Line 237…”C3 differed from C1 and C2 in all these time gaps” what is the basis for authors saying that there were differences in the time gaps? Is there any type of statistical comparison to support this?

Response: This is based on the Fisher information presented in Table 2. Fisher information closer to or above 1 means the variables are the determinants of cluster differentiation. This is principally very similar to ANOVA (with different analysis directions). ANOVA judges if the inter-group data variance is larger than the intra-group variance using pre-divided groups. On the other hand, cluster analysis categorises samples into different groups so that inter-group variance becomes larger than the intra-group variance, and Fisher information informs which variables are responsible for the clustering outcomes.

It is not appropriate to apply statistical testing for the group-based results because the aims of cluster analysis and hypothesis-driven statistics (such as ANOVA) are very different. Hypothesis-driven statistical tests aim to test the probability of the sample-based testing outcomes being applicable to the larger population. On the other hand, the cluster analysis is designed to separate the sample into sub-groups without considering the applicability of the outcome to the whole population, thereby it cannot provide any p-values (which essentially shows the probability of the outcome likely applicable to the whole population).

Nevertheless, due to the nature of the Fisher information calculation and cluster analysis results, those results are sufficient to back up the argument that C3 differs from C1 and C2 in the time gaps listed in the sentence.

Reference 20: invalid citation??

Response: Thank you for noticing the error. The reference has been replaced so that the citation shows the source clearly.

Lines 249-259 what can provide these observations from individual swimmers to the generalization of the present study results?

Response: It is about what “cannot” be generalised rather than what can be generalised. What these results show is that the cause of switching the coordination pattern is individually different and cannot be explained by a single factor. Such information is as equally important as suggesting generalisable results because “individually different” can also be a result that can be applied to a wide range of swimmers (L281-293).

Figure 3 contains tones of information. It may be split in two figures: one for AUW parameters and pull out phases and other for the time gaps. The same for table 3

Response: the figure and table have been divided into two as suggested (Table 4 and 5 and Figure 5 and 6).

At some point of the results section, it would be useful to present average absolute values of distance and times for each pullout subsection. This could serve as a frame of reference for coaches and practitioners.

Response: A table illustrating the absolute value of each time gap as well as 15 m and 25 m time has been included in the manuscript (Table 2).

Lines 303-313. If only four swimmers (less than half) modified their pullout pattern through the 200m event, then the second hypothesis could be partially rejected.

Response: The authors agree with the reviewer. However, this was the exact reason why the authors stated “partially supported” at the end of this section, which means the same as “partially rejected” (L357).

Lines 316-317 why the three coordination patterns were not tagged as in the mentioned previous publications (i.e. glide, continuity and superposition)?

Response: We did not use the same phrases as the previous study because we could not detect any continuity pattern. Both C1 and C2 were categorised within “glide” coordination, with a difference in the duration of the glide with arms at the side.

Line 338. “The differences between C1 and C2 were clearer in T1 and T6 than in T4 and T5” but in line 343-344 “T1 (duration of the glide with the arms at the side) was identical in these two groups”…isn’t this contradictory?

Response: There were some phrases missing in this sentence. The whole section has been rewritten for clarity, and with the new figure (Figure 3), the authors are confident the section became much clearer now (L382-390).

Reference #16 and #28 are the same

Response: Thank you for noticing the mistake. We have fixed this.

Line 351 Intra-individual variability section is probably too long, authors may consider splitting in two parts.

Response: The section has been divided into “Intra-individual variability”, “Systematic intra-individual variability” and “Summary and limitations”

Line 384 there is at least one more precedent in the literature that examined the underwater evolution on a 200m event in 25m pool

Response: Another reference that supports the argument has been inserted (reference #27).

Veiga, S., Mallo, J., Navandar, A., & Navarro, E. (2014). Effects of different swimming race constraints on turning movements. Human Movement Science, 36, 217-226.

Line 394-395 is there any precedent in the literature of a decrease in the glide time with fatigue?

Response: As far as the authors know, not for this particular glide motion (glide with the arms at the side). Olstad et al. (2020) showed that the push-off velocity decreased when 100 m breaststroke race progresses (which consequently affects the following glide velocity), but this is an explanation about the initial glide with the streamlined position – therefore it is not the best reference to support the argument. Nevertheless, fatigue is the likely explanation for this phenomenon and therefore the authors included a couple of phrases in this sentence (L438-439).

Lines 425-430 is there any limitation of the present study? This could be indicated here. For example, level of the participants (624 fina points) should be considered not even national level according to Ruiz-Navarro et al (2022)

Response: The limitation section has been included in the manuscript, and the description about the level of the swimmers has been modified (L469-494).

Lines 443-448 what was the role of the co-author AC?

Response: The role of AC was included in the descriptions “all authors contributed to…”. We have spelt all initials instead of “all authors” for clarity (L508-513).

Reviewer #2: General comments
I would like to congratulate the authors for the effort and time spent conducting this study, which aims, mostly, to investigate the intra- and inter-individual variability in arm-leg coordination during the underwater phase of the turn segment in 200 m breaststroke. This is an interesting study, under the scope of the journal and that could provide some practical applications for researchers, coaches and swimmers. The manuscript is well-written, easy to follow and with appropriate data analysis. These are the main strengths of the manuscript. Although the study is interesting, I have some concerns and below are my specific comments for each section.

Response: The authors appreciate the time the reviewer spent assessing our manuscript. The authors’ responses are all written in red font below. The line information provided in the responses all corresponds to the clean manuscript (not the track-changed version).

Abstract
- “LMM showed a decrease…”, please, be more specific (i.e., throughout the 200m trial?)

Response: This part of the sentence has been rephrased by “LMM showed a linear decrease (from the first to the last turn) in the time gap…” (L43-47)

- Should authors include that three patterns were found, in the conclusion?

Response: We have specified that the variabilities were categorised into three patterns. (L48-49)

Introduction
- Great work in the introduction. This is easy to read and understand the relevance of the study. Nevertheless, I think that it can be reduced to one or two paragraphs to be easier to follow. Moreover, some references should be added to support some sentences (for example, L60-61: 70-71; 117-120)

Response: References are not appropriate for the suggested sentences unfortunately. L60-61 (now L61-63) shows that there is little evidence in breaststroke underwater phase, which is not something literature can support. What we could potentially do is to list the number of articles that appeared on the literature search, but this is not reference-based evidence. L70-71 (now L71-72) is a general statement and an example with references is presented in L72-75. L118-121 is a summary section which basically describes a summary of what has been stated in the introduction – therefore the references that support this summary are all given in the sections before this statement.

- L117-123 The authors introduce here the concept of dynamic system theory and it is not clear in what way this could add something relevant to the introduction at this stage. I agree with a “summary” paragraph but struggled to understand this one. Please, this on this.

Response: Please note that all description about the theory is provided in an earlier section in the introduction and not in this summary section (L76-94). Assuming there is an “optimal” motion pattern means that the variabilities of the movement away from that optimal pattern are all considered to be undesirable noises. On the other hand, dynamic system theory states that variabilities are necessary for human to maximise their performance as constrains with which they generate movements constantly change (fatigue, energy resource, environmental changes etc.), meaning that an optimal technique under one condition is not optimal under other conditions and therefore human should keep adapting their movements to maximise their performance – hence the variability is generated for the functional purpose. The purpose of this section is to emphasise the importance of investigating the variability rather than seeking one optimal solution.

Methods
- Very nice section. Congratulations. Some minor comments to consider. i) The warm-up was individual but was there any assessment or control? Could 20-30 min of rest after warm-up be too much (according to the literature, this is enough to reduce the warm-up effects)?

Response: The reason for this rest time is that swimmers usually have to wait at least 20-30 mins (sometimes even longer) in real races (changing swimsuits, heading to the call room, etc.). The authors tried to make the swimmers’ condition as close to the real race condition as possible rather than aiming to prepare their condition as best as possible, hence the time lag between the warm-up and the time trial.

- The swimmers competed alone in the swimming pool. I understand this is the appropriate procedure for this. However, this does not “mimic” a competition situation. Could this influence results? Could the real competition setting influence pattern? Please, this should be included in the discussion section/limitation section.

Response: Three limitations are included at the end of the discussion (L469-494).

- The authors should change “200m race” to “time-trial”, as this is more appropriate for the testing procedures that were used.

Response: Throughout the manuscript, “200 m race” has been replaced with “200 m trial”.

- Each swimmer performed a 200 m time trial once. There is a day-to-day variability that we should acknowledge in the discussion. To be more accurate, I think authors should replicate the 200m time trial and calculate reliability measures. This would support the main findings.

Response: The potential effect of the day-to-day variability is listed in the limitation section (L478-479). On the other hand, however, it is unlikely that repeating the 200 m trials would change the main outcomes of the study. For those swimmers who maintained one single pattern throughout the trial, it is reasonable to assume that that coordination is their typical pattern – which is unlikely changed to the other pattern even if the trial is repeated on a different day (if they do change the pattern, then that should be considered that the swimmer completely changed their technique, rather than it shows a day-to-day variability). What could be affected by repeated trials would be the coordination patterns of those who switched the coordination in the middle of the trial (e.g. at which point of the trial they changed the coordination). However, it is a matter of the timing rather than the technique they adapt. Therefore, one of the main outcomes of the study (there are three coordination patterns) would not be affected much. Another main outcome of the study was the systematic reduction in the two time-gaps and the total underwater phase. Assuming that the main explanation of these phenomena is fatigue, this effect should still exist whenever swimmers perform under whatever conditions.

- By using the swimmer's head to determine horizontal velocity instead of hip centre/body centre, could this conditioned results?

Response: It is unlikely that this would severely affect the outcomes. If the other body part was used instead of the head, then the variable value would certainly be different. However, this change should be systematic and having a systematic change in the variable values should not affect either the cluster analysis or the mixed modelling because both analyses investigate the variability within each variable (and the systematic change would not affect the variability).

- There is no data regarding the times performed during the trial and how different they were from each personal best. This information should be added as it can be important to validate results.

Response: The total time information has been included at the beginning of the result section (L243).

- Why did the authors choose to use those specific covariates? For example, lap as a fixed effect and the participant as a random effect?

Response: Mixed modelling analyses the data using the equation y = a + bt, where a refers to the random effect, b is the fixed effect, and t is the different time/condition, meaning that b is the coefficient that affects the outcome depending on time/condition and a is the random variability. When applying 13 participants' data x 7 turns (corresponding to the condition), considering the participant effect as a and the turn effect as b would be the most reasonable, as the other way around is clearly illogical because the participants are not the condition, different laps are.

Results
- Perhaps I did not understand well, but, why the authors did not include the initial glide in the analysis.

Response: the initial glide phase is a passive motion and does not fit the definition of active underwater motion, meaning that it does not answer the main question of the present study (i.e. inter-limb coordination). This is the reason why the authors did not include the initial glide phase in the cluster analysis.

- Figure 3 is important and should be highlighted.

Response: This figure (now Figure 5 and 6 in the current version) is highlighted in the result and detailed interpretation is discussed in the ‘Systematic intra-individual variability’ section in the discussion.

- In my opinion, Figure 4 does not add anything relevant to this study. However, if the authors choose to include this, this should be included in the results and removed from the discussion.

Response: Figure 4 and related sentences have been excluded from the manuscript.

- There is no information about the 200 m times. This should be included in the results.

Response: The definition of each cluster was based on each lap rather than the whole 200 m performance, so unfortunately, it is not possible to use the 200 m time as an indicator of each cluster performance. Instead, the authors have included 15 m and 25 m time in each lap as the performance indicator (Table 2).

Discussion
- The authors analysed swimming patterns and found different patterns (n = 3). However, the information about how this influences performance is not available and, in my opinion, is the major gap in this study. There is no information about the performance (total performance or laps) and if there was there any relationship between the higher level swimmers with some specific pattern. I think the authors should improve the practical application of the findings, perhaps by including some discussion/analysis based on this.

Response: We have included the 15 m and 25 m time as the performance indicator (Table 2). It is difficult to discuss if one cluster is better than the other, as some swimmers selected C2 in the latter stage of the trial, which means that these laps are likely slower than at the beginning of the trial due to the fatigue effect. However, we did include some discussion based on the difference in the 15 m time (in relation to the 25 m time) in the discussion section (L415-420).

- Could the authors provide some reason for the only swimmer that used a completely different pattern?

Response: At this point, it is difficult to make any argument as only 1 swimmer showed this pattern, meaning that it is not possible to differentiate the individual characteristics and mechanisms behind this particular coordination pattern.

- Please remove the results from the discussion (Figure 4). The discussion should not provide new results to the manuscript.

Response: The figure and related sentences have been excluded.

- L425, please be careful. I think that the time and FINA points do not correspond to elite-level swimmers.

Response: The description of the swimmers’ level has been modified (L492).

- Considering your thoughts and these comments, please include limitations in the discussion section.

Response: Three limitations have been included at the end of discussion section (L478-494).
