# Supplementary material for: Intra- and inter-individual variability in the underwater pull-out technique in 200 m breaststroke turns
Source: PLoS One. 2023 Mar 21;18(3):e0283234. doi: 10.1371/journal.pone.0283234 (PMC10030011; doi:10.1371/journal.pone.0283234)
Supplement: Attachment — Submitted filename: Reviewer_comments_responses_R2.docx [file pone.0283234.s004.docx]

Reviewer #1: Authors responded adequately to the majority of points indicated by reviewer in the first revision. However, there are still some points that should be further revised:

- Authors modified the naming of the different time gaps and this undoubtedly will help readers to better understanding data. However, for the present reviewer (and this is only an opinion), it would be easier if key events were named in the order they usually occur during breaststroke pullout. For example, in T4 DKend > APObeg instead of APObeg - DKend
Or in T6 DKend >LRbeg instead of LRbeg – Dkend

The name has been edited by changing the order of the key points.

- A selection of real swimming frames containing the typical key events and swimmers positioning in the three different clusters would be much more intuitive and clear for readers than figure 3.

The figure has been updated accordingly.

- Considering the large extension of results section, could table 2 presented as supplemental material (appendix)?

Thank you for the suggestion. We discussed this point among the authors but concluded that this table would probably be better to be shown in the result section, as the authors discuss the absolute data in the discussion section (albeit not to a large extent; L415-420, corresponding to L427-433 in the track-changed version) and also the other reviewer strongly suggested to include the absolute time information.

- Should tables 4 and 5 contain any additional explanation of the statistical effect presented with the F ratio and p-value? Do they refer to the lap effect, right? Why authors do not explicitly indicate it?

We appreciate the suggestion from the reviewer. The captions for the two tables were edited so that it clearly indicates that the values in the tables all show lap effects.

- Could be the decrease in the glide time and distance displayed through the 200m turns (like for example in T1) be interpreted in line with decrease in non-propulsive time gaps during breaststroke swimming (ie. Chollet, D., Seifert, L., Leblanc, H., Boulesteix, L., & Carter, M. (2004). Evaluation of arm-leg coordination in flat breaststroke. International journal of sports medicine, 25(07), 486-495) or in 100m freestyle races (Seifert, L., Boulesteix, L., Carter, M., & Chollet, D. (2005). The spatial-temporal and coordinative structures in elite male 100-m front crawl swimmers. International Journal of Sports Medicine, 26(04), 286-293)?

We thank the reviewer for this suggestion. We have included some descriptions about the possibility of swimmers compensating for the loss of performance by minimising the glide phase (L439-441 in the clean version, L455-458 in the track-changed version). We, however, have not included references for this explanation as it is debatable how similar front crawl/breaststroke (both cyclic movements) and underwater pull-out (closed/isolated skill) are.
