# Supplementary material for: Intra- and inter-individual variability in the underwater pull-out technique in 200 m breaststroke turns
Source: PLoS One. 2023 Mar 21;18(3):e0283234. doi: 10.1371/journal.pone.0283234 (PMC10030011; doi:10.1371/journal.pone.0283234)
Supplement: Attachment — Submitted filename: reviewer comments 2.docx [file pone.0283234.s003.docx]

Authors responded adequately to the majority of points indicated by reviewer in the first revision. However, there are still some points that should be further revised:

- Authors modified the naming of the different time gaps and this undoubtedly will help readers to better understanding data. However, for the present reviewer (and this is only an opinion), it would be easier if key events were named in the order they usually occur during breaststroke pullout. For example, in T4 DKend > APObeg instead of APObeg - DKend

Or in T6 DKend >LRbeg instead of LRbeg – Dkend

- A selection of real swimming frames containing the typical key events and swimmers positioning in the three different clusters would be much more intuitive and clear for readers than figure 3.

- Considering the large extension of results section, could table 2 presented as supplemental material (appendix)?

- Should tables 4 and 5 contain any additional explanation of the statistical effect presented with the F ratio and p-value? Do they refer to the lap effect, right? Why authors do not explicitly indicate it?

- Could be the decrease in the glide time and distance displayed through the 200m turns (like for example in T1) be interpreted in line with decrease in non-propulsive time gaps during breaststroke swimming (ie. Chollet, D., Seifert, L., Leblanc, H., Boulesteix, L., & Carter, M. (2004). Evaluation of arm-leg coordination in flat breaststroke. *International journal of sports medicine*, *25*(07), 486-495) or in 100m freestyle races (Seifert, L., Boulesteix, L., Carter, M., & Chollet, D. (2005). The spatial-temporal and coordinative structures in elite male 100-m front crawl swimmers. *International Journal of Sports Medicine*, *26*(04), 286-293)?
